# Supplementary material for: Hematological and immunological profiles of podoconiosis patients in West Gojjam Zone, Ethiopia: A comparative cross-sectional study
Source: PLoS Negl Trop Dis. 2024 Dec 17;18(12):e0012732. doi: 10.1371/journal.pntd.0012732 (PMC11687886; doi:10.1371/journal.pntd.0012732)
Supplement: Attachment — Submitted filename: Article Reviewers Comments- Atnaf.docx [file pntd.0012732.s001.docx]

**REVIEWERS COMMENTS**

1. Abstract

Please elaborate more on why you examined stool samples in your study. Are there any findings / results of stool samples mentioned in the abstract? Please note that your abstract should not exceed standard word limit as per this journal and must only include the following separate sections:

2. Conclusions/Significance: Please separate the two, and follow the standard Abstract format for this journal.
3. Introduction: This part lacks information on Hematological evidence, please have information in regard to hematology.
4. Methods: Please bring more information on selection criteria, seems not coming out well. What is the clinical relevancy of ABO & Rh in podoconiosis, Malaria? and stool analysis?

What do you tell a lay man on the role of Immunological evidence using those cytokines? Please lets bring out well, remember this is a laboratory based study.

On blood samples: what do you mean when you say blood left overs centrifuged???, lets be scientific please.

How did you handle waste management? And quality control / assurance on other assays apart from CBC?

5. Ethical approval: Consent to participate

   In your ‘Ethics approval and consent to participate’ section, please clarify whether informed consent / assent to participate was obtained from all of the participants in the study and clearly state this in on how you managed it especially minors. As such, we ask that in your ‘Ethics approval and consent to participate’ section, to clarify whether informed consent to participate, written or verbal, was obtained from the parents or legal guardians of any minor participant.
6. Limitations

   Please provide a separate Limitations section, seems not clear.
   Authors must clearly state any limitations they see in relation to their data. Limitations may be presented in bullet point format.

Thank you.
