# Supplementary material for: Hematological and immunological profiles of podoconiosis patients in West Gojjam Zone, Ethiopia: A comparative cross-sectional study
Source: PLoS Negl Trop Dis. 2024 Dec 17;18(12):e0012732. doi: 10.1371/journal.pntd.0012732 (PMC11687886; doi:10.1371/journal.pntd.0012732)
Supplement: Attachment — Submitted filename: Response to Reviewers.docx [file pntd.0012732.s002.docx]

**Response to Reviewers**

**Manuscript ID: PNTD-D-24-00819) - [EMID:8c8c9c1d99ba1451]**

**Hematological and Immunological Evidence for Podoconiosis Autoimmune Disease Pathogenesis, West Gojjam Zone, Ethiopia: A Comparative Cross-Sectional Study**

**Dear Reviewers and Editor**,

We would like to express our gratitude for the time and effort you dedicated to the rigorous review of our manuscript. We have carefully considered each of your comments and suggestions and have made corrections accordingly.

In the revised manuscript, we have tracked the changes using blue text to make them easily identifiable. In this point-by-point response document, we have provided our replies in blue for clarity and indicated using page and line numbers for amendments we did in the manuscript.

We hope that the revisions address your concerns and improve the quality of the manuscript. Thank you again for your valuable feedback and for guiding us through this process.

**Reviewer #1:** The objective of the article clearly articulated.

The study design has a detailed procedure on the laboratory evaluation but the authors failed to mention on how the diagnosis and clinical staging is done, how the sampling is calculated (What was the logic behind recruiting 53 podoconiosis patients and 67 controls), how is podoconiosis diagnosed and who has diagnosed them. Additionally, how is the staging done (mild, moderate and severe) and the criteria used is not clear. Taking the multiple variables comparison to consideration the very small sample size of patients (5 mild, 21 moderate, 27 severe) is clearly one major critical limitation of the study. The small sample size and the variation of the patient staging makes it insufficient to ensure adequate power for the hypothesis being tested.

The author stated the ethical approval for the study is secured from the regional health bureau (which as far as my knowledge goes the bureau does not have a committee....they only provide support letter), while the authers affiliation is in the university (and it is known both Debremarkos University and AAU have IRB process (mandated by national ethical committee). Most importantly the author claims the data is collected in the IOCC podoconiosis treatment centers but no one from the NGO is mentioned as co-author.

**Authors’ responses:**

**Dear Reviewer,**

Thank you for your detailed comments and concerns regarding our manuscript. We are truly grateful to receive suggestions from such a senior expert in podoconiosis, and we feel fortunate to have your guidance in improving our work.

**Diagnosis and Clinical Staging of Podoconiosis**:

The diagnosis and clinical staging in our study were conducted by a trained Public Health expert at the IOCC center and a Physician, following the five-stage system described by Tekola F. et al. (2008). We have clarified this in the revised manuscript page 8, line 132-143.

**Sample Size Calculation and Recruitment:**

For the sample size calculation, we used Epi-info software, assuming a 90% confidence level, 80% power, and a ratio of 1.26 healthy controls to patients. We also used the mean and standard deviation of hemoglobin concentration for patients (13.7 ± 2.29 g/dl) and healthy controls (14.7 ± 2.04 g/dl) from Taye B. et al. (2013). This yielded a total sample size of 120, consisting of 53 podoconiosis patients and 67 healthy controls. We have now included this explanation on page 9, lines 144-150, as per your suggestion.

**Staging of Lymphedema:**

Regarding the severity classification during data analysis, we categorized stage 1 as ‘mild,’ stage 2 as ‘moderate,’ and stages 3, 4, and 5 as ‘severe,’ given that these advanced stages represent irreversible lymphedema. We made these classifications to align with the clinical understanding of disease progression.

**Ethical Approval:**

We obtained ethical approval from both the Amhara Health Bureau and Addis Ababa University (AAU). Initially, we included only the approval from the Amhara Health Bureau, as we believed one approval was sufficient. However, we have now also included AAU’s ethical approval on page 23, lines 464-467.

Concerning your comment about the Amhara Health Bureau not having an ethics committee, we would like to clarify that, in 2017, the Bureau did indeed have a committee responsible for granting ethical approval. Currently, this responsibility has shifted to the Amhara Public Health Institute (APHI). We possess an ethical approval letter from the Health Bureau, and we are happy to provide this document as evidence to the editor, should further clarification be required.

We appreciate your insightful feedback and have addressed your concerns to the best of our ability in the revised manuscript.

Reviewer #1: The result is presented according to the analysis plan and result is completely presented.

**Authors’ responses:**

**Dear Reviewer,**

We are grateful for your positive feedback. It motivates us to continue creating high-quality work.

Reviewer #1: In general, the conclusion is not supported by the finding and the author bold statement mentioned starting on the title are based on false premiss. The limitation of the study such as the small sample size, unclear diagnostic and staging criteria and the fact all the authors are laboratory personals with no clinical background for participant recruitment is not mentioned.

**Authors’ responses:**

Dear Reviewer, we sincerely thank you for your valuable comments and feedback.

**Regarding the Autoimmune Nature of the Disease:**

We have provided evidence supporting the autoimmune nature of the disease based on our findings, as well as supportive references. Therefore, we respectfully assert that the title of our manuscript is not based on a false premise.

**Authors' Background:**

In response to your concern about the authors’ background, our team is composed of professionals from various disciplines, including immunology, microbiology, hematology, biology, and clinical epidemiology. While some of the authors are laboratory professionals, this interdisciplinary mix ensures a comprehensive approach to the research. Moreover, we collaborated with a clinical expert from IOCC and a physician to gather clinical data from patients, including diagnosis and staging.

**Participant Recruitment:**

Regarding participant recruitment, we selected podoconiosis patients and healthy controls who met the inclusion criteria outlined in the manuscript (Method section, paragraph 2, lines 132-143). However, we acknowledge that we did not clearly describe the recruitment technique in the original manuscript. Based on your valuable comment, we have now inserted the details of the recruitment process into the revised version.

**Limitation of the Study:**

The limitations of our study were previously included in the discussion section, but to avoid any confusion, we have now added a clear subheading titled “Limitations” in the discussion (page 21, lines 421-435).

We appreciate your thorough review and have made the necessary revisions to improve the manuscript.

Reviewer #1: The title is very bold statement to attract the attention of experts who are working on the disease but when I dive deep to findings it does not have the evidence to support the title. 
The authors look does not have much experience/knowledge on podoconiosis which is apparent to see in the introduction wrong conclusions like  ‘ Podoconiosis classified as a Neglected Tropical Disease 45 (NTD) by the World Health Organization in 2011’  which is not right statement …..podo is not added under the list of WHO NTD list. And conclusive statements like “Podoconiosis, ….is a geochemical disease caused by alkali volcanic-originating soil’  ...this is so far a hypothesis (the cause effect relation of podoconiosis and soil is not confirmed) needs additional study to conclude.

**Authors’ responses:**

**Dear Reviewer,**

We sincerely thank you for your concerns regarding the introduction of our manuscript. While we acknowledge that our experience with podoconiosis may not be as extensive as yours, we did refer to a specific article that mentions podoconiosis being included in the WHO list of skin NTDs as of February 2011. One of our coauthors, Professor Aster, is also a coauthor of that particular article. For your reference, you can review the publication here:
<https://pubmed.ncbi.nlm.nih.gov/22423883/>.

Upon checking the WHO website, we understand there is no direct statement about podoconiosis being included in the list of NTDs, but it is listed under "skin NTDs." With due respect, if this article turns out to be incorrect, we will amend our statement accordingly.

**Regarding the Statement on the Geochemical Cause of Podoconiosis:**

We appreciate your concern about our phrasing of podoconiosis as a "geochemical disease caused by alkali volcanic-originating soil." We agree with your point that this remains a hypothesis and needs further study to confirm a cause-effect relationship between podoconiosis and soil exposure.

In our manuscript, we mentioned in paragraph 2 of the introduction (page 5, lines 79-85): *“A geochemical study linked specific minerals (smectite, mica, and quartz) in the soil with a higher prevalence of podoconiosis, suggesting that some feature of these non-organic soil particles may have a role in disturbing the lymphatic systems of susceptible people.”* Therefore, this issue was not a result of a lack of knowledge or experience, but rather a transcription error in the initial draft. In the final manuscript, we have amended the statement to read: *“Podoconiosis is* ***suggested as*** *a geochemical disease caused by alkali volcanic-originating soil”. We added the word “suggested as” in the statement in page 5, line 62.*

We thank you again for your valuable feedback, which has greatly improved the clarity and accuracy of our manuscript.

Reviewer#1: Study Findings and Conclusion

The conclusions of the study are not supported by the findings. The limitations of the study, such as the small sample size, unclear diagnostic and staging criteria, and the lack of clinical expertise among the authors, are not adequately addressed. The discussion attempting to link podoconiosis to autoimmune disease lacks scientific grounding.

**Authors’ responses:**

**Dear Reviewer,**

Thank you for your concerns regarding the conclusions of our findings. We appreciate your thorough review and would like to clarify that our conclusions are supported by evidence. We have added more information to the manuscript to ensure the conclusion is clearer.

1. **Silica Exposure and Autoimmunity:** Animal models have shown that silica exposure can lead to the development of autoimmune diseases, particularly in the lungs.
2. **Abnormal Neutrophil Counts:** In our study, we observed abnormally low absolute neutrophil counts, which may be associated with autoimmune neutropenia (AIN), a condition where autoantibodies target and destroy neutrophils in the patient.
3. **High Lymphocyte Counts and Hemoglobin Concentration:** We also found abnormally high lymphocyte counts in patients, along with reduced hemoglobin concentration. This reduction may be linked to autoimmune destruction of red blood cells, although this aspect of the disease requires further investigation.

While we believe our findings indicate potential autoimmune involvement, we agree that the exact autoimmune nature of the disease needs more in-depth research. We have clearly stated this as a limitation in our study, and to avoid any confusion, we have provided a section titled “Limitations” in the manuscript.

Regarding your concern about the “lack of clinical expertise among the authors,” we respectfully disagree. Several of the authors have a clinical background and have previously published work on podoconiosis. Moreover, the clinical staging and diagnosis of the disease in our study were carried out by a trained expert from IOCC, with support from a physician who was compensated for their services. We have specified this information on page 8, lines 132-143 of the manuscript for clarity.

Thank you once again for your valuable feedback, which has helped us improve the clarity and robustness of our study.

Specific issues include:

1.      Hematologic Profiles: The study indicates that podoconiosis patients have decreased hematologic profiles compared to controls, but these values remain within normal ranges, suggesting that these patients are not pancytopenic. A reduced hematologic profile can result from various conditions such as malnutrition, drugs, infections, or malignancies, not exclusively autoimmune diseases.

**Authors’ responses:**

Dear Reviewer,

Thank you for your insightful comments. We would like to clarify a few points regarding the hematological findings.

1. **Hematological Cell Counts:** Not all hematological cell counts were within the normal range. For example:
   - **Absolute neutrophil counts** were decreased.
   - **Eosinophil counts** were decreased.
   - **Lymphocyte and monocyte counts** were increased.

Additionally, the mean **hemoglobin concentration** was below the normal range. While pancytopenia is commonly associated with autoimmune diseases, it is not a universal finding. In autoimmune conditions like **Rheumatoid Arthritis**, elevated circulating T cells can lead to increased lymphocyte counts, which in turn may raise the total WBC count. For reference, please see this study: <https://pubmed.ncbi.nlm.nih.gov/30809737/>.

2. **Autoimmune Disease and Confounding Variables:** While the reduced cell counts may not necessarily be specific to autoimmune disease and could be influenced by underlying conditions, we took measures to minimize confounding variables. Both podoconiosis patients and healthy controls with known chronic diseases were excluded from the study to ensure more accurate results.

We hope this clarifies our findings and the rationale behind our interpretations. Thank you once again for your valuable feedback.

2.      Other Causes: The study does not rule out other potential causes for the decreased hematologic profiles in podoconiosis patients, such as hookworm-induced anemia due to barefoot exposure, which the study acknowledges as common among podoconiosis patients.

**Authors’ responses:**

Dear Reviewer,

Thank you for highlighting this critical issue. Your comments have been extremely valuable for improving our manuscript.

We would like to clarify that we did use exclusion criteria to rule out other chronic illnesses, such as HIV, TB, DM, and HTN. In addition, we performed hemoparasite and intestinal parasite (IP) screenings to rule out parasite-induced anemia. We employed wet mount and concentration techniques to examine intestinal parasites.

To further investigate the cause of anemia, we conducted a **univariate analysis** of hemoglobin (HGB) concentration as an outcome (dependent variable), with disease status and hookworm infection status as factors. Our analysis yielded the following results:

- Hookworm infection did not cause a statistically significant variation in HGB levels among study participants (F = 1.39, p = 0.166).
- However, **podoconiosis status** significantly impacted HGB levels (F = 42.33, p < 0.0001). The mean HGB level of podoconiosis patients was significantly lower than that of healthy controls.
- The mean HGB level of **podoconiosis patients co-infected with hookworm** (11.69 g/dl ± 0.96) and **podoconiosis patients without hookworm** (12.21 g/dl ± 0.90) showed no statistically significant variation (F = 0.061, p = 0.805).
- The mean HGB level of cases (12.06 g/dl) was significantly lower than that of controls (14.14 g/dl), and this difference was statistically significant (F = 43.32, p < 0.001).
- For controls infected with hookworm, the mean HGB level was 13.82 g/dl, which falls within the normal reference range (12–18 g/dl for females, 13.6–19.8 g/dl for males).

These findings suggest that anemia in podoconiosis patients may not be due to hookworm infection but rather linked to podoconiosis itself.

However, we chose not to include this detailed parasitological analysis in the current manuscript, as we plan to publish these findings in a separate paper focusing on parasitological investigations.

We appreciate your thoughtful feedback and look forward to further improving our work based on your valuable input.

3.      Contradictory Findings: The report that absolute lymphocyte and monocyte counts are significantly higher in podoconiosis patients contradicts the notion of pancytopenia and does not support the autoimmune disease hypothesis.

**Authors’ responses:**

Dear Reviewer,

Thank you for your insightful comments. We acknowledge that different autoimmune diseases can present with varying hematological profiles, such as reduced, normal or increased counts of WBCs, platelets, neutrophils, lymphocytes, or RBCs. For example, bronchial asthma, which is classified as an autoimmune disease, is characterized by elevated lymphocytes, mast cells, and eosinophils ([PubMed](https://pubmed.ncbi.nlm.nih.gov/17896974/)), SLE disease has elevated lymphocytosis due to hyperactivation of the B lymphocytes.( <https://arthritis-research.biomedcentral.com/articles/10.1186/ar3433>). On the other hand, Rheumatoid arthritis (RA) patients may sometimes experience an increased or decreased platelet count during active inflammation, many patients maintain a normal platelet count. ([Johns Hopkins Arthritis Center](https://www.hopkinsarthritis.org/arthritis-info/rheumatoid-arthritis/ra-symptoms/) Stat care [Very well Health](https://www.verywellhealth.com/platelet-counts-in-rheumatoid-arthritis-189614))

We have included two of the above references in the manuscript on page 19, lines 366-367.

Therefore, autoimmune disease does not always manifest by pancytopenia.

Given these variations, our study suggests that podoconiosis might have an autoimmune component, but we concur that definitive conclusions regarding its autoimmune nature require further detailed investigation.

Thank you for highlighting this important aspect of our research.

4.      Immunologic Profiles: The observed reduction in TNF-α among podoconiosis patients contradicts the argument for an autoimmune disease. The decrease in CD4 count, proposed as evidence for autoimmune disease, overlooks the fact that in humans, CD4 depletion (as seen in HIV infection) usually ameliorates clinical activity rather than exacerbating it.

**Authors’ responses:**

Dear Reviewer,

Thank you for your thoughtful questions and comments. We appreciate the opportunity to address them.

The observed reduction in TNF-α levels among podoconiosis patients was not statistically significant. One possible explanation for this finding is the relatively small number of early-stage cases included in our study. It is known that in advanced stages of podoconiosis, there tends to be an increase in anti-inflammatory cytokines rather than pro-inflammatory cytokines. Additionally, since controls are also exposed to red clay soil, their immune systems might interact with the soil components, potentially leading to the release of various cytokines. However, individuals with podoconiosis may have heightened susceptibility to these effects.

We agree that further research is needed, particularly studies including controls who are both unexposed to the soil and unaffected by the disease. This will help to clarify the relationship between TNF-α levels and podoconiosis more definitively.

Thank you once again for your valuable feedback.

5.      Silica Association: The claim of an association between podoconiosis and silica is not supported by recent geochemical studies, which show no significant correlation. The mean silica particle concentration is slightly higher in non-endemic areas for podoconiosis, refuting the hypothesis of silica involvement. (actually the mean silica particle concentration is slightly higher in non podoconiosis endemic area) (Cooper, Jamey N, and Kevin E Nick. “A geochemical and mineralogical characterization of soils associated with podoconiosis.” Environmental geochemistry and health vol. 45,11 (2023): 7791-7812.)

**Authors’ responses:**

Dear Reviewer, we thank you for providing us recent updates. We are sorry for missing it to include in our manuscript. Our research was conducted before this publication but we need to include it. Currently, we incorporated this article in the introduction part (Page 5-6, line number 79-85) to provide recent updates to the readers. Thank you for your valuable comments.

Summary
The title of the study is misleading, and the conclusions drawn do not align with the actual findings. The study's significant methodological flaws and unsupported claims undermine its contribution to the understanding of podoconiosis. The authors need to address these limitations and provide a more balanced and accurate interpretation of their data.

**Authors’ responses:**

Dear Reviewer, thank you for providing us a summary of your comments. We believe our title is not misleading because of our pertinent findings that could be related to autoimmunity. We have addressed the specified limitations.

Reviewer #3: The article is generally interesting, as there is still much to be learned about podoconiosis. The objectives are clear and the research is well developed, although, as the authors state at the end of the manuscript, proper footwear and education are the pillars of prevention and treatment, so in the future it may be more useful to address the next steps in this direction. The study design is adequate to address the stated objectives and the population is clearly described for the hypothesis. However, there are still data that could be more detailed. As the authors explain in the discussion, "low hemoglobin levels reflects the hemolysis that occurs through chronic inflammation, as additionally seen with RBCs significantly decreasing by stage from mild to advanced". In this regard, for the study of anemia, if present, LDH and total bilirubin should have been studied as the presence of hemolytic anemia is of concern. The sample size should be increased in future investigations, but enough to ensure adequate power to address the hypothesis being tested. The statistical analysis is correct and there are no ethical or regulatory concerns.

**Authors’ responses:**

Dear Reviewer, we would like to appreciate your interesting insights in to the manuscript. We have accepted your suggestions with due respect for future research works. We will consider the test parameters you mentioned for future research investigations with an adequate sample size.

Reviewer #3: The analysis match the analysis plan. Results are clearly and completely presented and figures are of sufficient quality for clarity. Nonetheless, regarding age, cases were aproximately 8 years older than controls (49 vs 41yo). As far as we know, direct contact for a longer period (age) increases the risk for podoconiosis. Also age could increase the risk for other diseases and anemia due to other causes such as cancer. Authors should specify comorbidity or concomitant illnesses of the studied population. Anemia could also be explained by the presence of parasites (hookworm and other soil-transmitted helminths). In the table, it is described the presence of "any parasite" in 37,7% of cases and 32,8% of controls. Even if no statistically significant, authors should describe what parasites they found in stools or other tests.
Additionally, it is remarkable that 24% of cases (13p) declared washing their legs twice a day vs. 6% (4p) of the controls. How do the authors explain this unexpected finding?

**Authors’ responses:**

Dear Reviewer,

Thank you for your valuable comments.

We agree that podoconiosis typically requires a minimum of 10 years of direct soil contact to develop, which was a key inclusion criterion for our study participants. The mean age difference of 8 years between patients and healthy controls might influence the results, as aging can affect immunity and increase susceptibility to infections, anemia, malignancy, etc. While we implemented stringent exclusion criteria for chronic diseases (cancer, CKD, CLD, TB/HIV), we did not use a sex and age-matched study design. Consequently, the age variation is expected.

Regarding parasitic infection-induced anemia, our univariate analysis revealed that parasitic infections did not significantly contribute to low hemoglobin levels, whereas podoconiosis was significantly associated with anemia (p < 0.05). Most of our podoconiosis patients (88.7%) wore shoes to protect their feet from soil exposure, which likely reduces the risk of hookworm infection—a major cause of helminth-induced anemia. We have chosen not to include these detailed findings in the current manuscript as they will be presented in a separate publication focusing on parasitological investigations.

Regarding leg-washing practices, our data showed that podoconiosis patients wash their legs more frequently than controls. This increased frequency is likely due to the need to alleviate lymphedema-related discomfort. Frequent washing helps reduce pain and is also practical for patients who are less involved in physically demanding activities, such as farming, due to their condition.

Thank you again for your insightful feedback, which has greatly contributed to the refinement of our manuscript.

Reviewer #3: Conclusions are supported by the data presented and limitations are clearly described.

**Authors’ responses:**

Dear Reviewer, we thank you for showing telling the fact we have made the correct conclusion and limitation of the manuscript. It provides internal power to us to improve the quality of our work.

**REVIEWERS COMMENTS (from word document)**

1. Abstract

Please elaborate more on why you examined stool samples in your study. Are there any findings / results of stool samples mentioned in the abstract? Please note that your abstract should not exceed standard word limit as per this journal and must only include the following separate sections:

**Authors’ responses:**

Thank you for highlighting the issue regarding the stool sample examinations. We collected stool samples from both podoconiosis patients and healthy controls to investigate the presence of intestinal parasites that might contribute to anemia. This was done to determine whether the anemia observed was due to intestinal parasites, podoconiosis itself, or a combination of both. We did not include the results of the stool sample examinations in the abstract or the main text of this manuscript because the findings are part of a broader project involving multiple laboratory investigations. We are preparing a separate manuscript to detail the parasitological results and their implications. We appreciate your understanding and patience as we work on presenting these findings comprehensively in another publication.

Thank you again for your valuable feedback.

2. Conclusions/Significance: Please separate the two, and follow the standard Abstract format for this journal.

**Authors’ responses:**

Dear Reviewer, we used the PLOS NTD manuscript submission guideline and it stated merging the two in one. So, we did it according to the guidelines.

3. Introduction: This part lacks information on Hematological evidence, please have information in regard to hematology.

**Authors’ responses:**

Dear Reviewer, thank you for your critical comments. At the time of manuscript preparation, there was no hematological evidence of podoconiosis published. However, currently, we have found only one article and included hematological information in the introduction part based on your comment in page 6, line 96-100.

4. Methods: Please bring more information on selection criteria, seems not coming out well.

What is the clinical relevancy of ABO & Rh in podoconiosis, Malaria? and stool analysis?

What do you tell a lay man on the role of Immunological evidence using those cytokines? Please lets bring out well, remember this is a laboratory based study.

On blood samples: what do you mean when you say blood left overs centrifuged???, lets be scientific please.

How did you handle waste management? And quality control / assurance on other assays apart from CBC?

**Authors’ responses:**

Dear Reviewer,

Thank you for your insightful questions and comments.

1. **Selection Criteria:** We have now detailed the selection criteria of study participants in the manuscript as per your suggestions. Please refer to page 8, lines 132-143 for the updated information.
2. **Blood Grouping and Screening:** The blood grouping was conducted to investigate any potential variation between different blood groups and to explore possible genetic susceptibility within families. We also screened for malaria and intestinal parasites (IP) to identify potential confounders of anemia, as both can contribute to anemia and may overlap with the anemia associated with podoconiosis.
3. **Immunological Evidence:** Cytokines are critical in studying immune responses. They are small proteins produced by immune cells that regulate inflammation. Pro-inflammatory cytokines promote inflammation, while anti-inflammatory cytokines facilitate fibrosis. In laboratory studies, cytokines help elucidate the immune system's role in diseases by indicating the nature of the immune response. In simpler terms, cytokines are molecules that assist the immune system in fighting infections and maintaining balance. We have added the definition of cytokines on page 11, line 190-193.
4. **Clarification on "Leftover":** The term "leftover" we mean the remaining blood sample in the EDTA test tube. We have made changes to this terminology on page 10, line 166 for clarity.
5. **Waste Management and Quality Assurance:**
   - **Waste Management:** We segregated wastes into three categories—infectious, sharp, and non-infectious—before disposal by incineration at the healthcare facilities (hospital and two health centers).
   - **Quality Assurance:**
     - We employed blank and standard for cytokine ELISA measurements.
     - Quality control was performed for CD4 counts.
     - Known blood group individuals were used for anti-sera (anti-A, anti-B, and anti-D) quality control testing.
     - Giemsa reagent quality was assessed using malaria-positive and -negative slides.
     - Fresh saline, formalin, and ether were used for intestinal parasite wet mount and concentration techniques.
     - We adhered to the standard operating procedures throughout all phases of quality assurance: pre-analytical, analytical, and post-analytical.

We appreciate your feedback, which has significantly contributed to improving the quality and clarity of our manuscript.

Thank you for your consideration.

5. Ethical approval: Consent to participate

   In your ‘Ethics approval and consent to participate’ section, please clarify whether informed consent / assent to participate was obtained from all of the participants in the study and clearly state this in on how you managed it especially minors. As such, we ask that in your ‘Ethics approval and consent to participate’ section, to clarify whether informed consent to participate, written or verbal, was obtained from the parents or legal guardians of any minor participant.

**Authors’ responses:**

Dear Reviewer, thank you for your concern on Ethical approval section of consent to participate. However, our study participants were adults with age 18 years and above as shown in the inclusion criteria. So, we obtained written informed consent from them for participation in the study. We narrated in the document you can recheck it from page 9 line 152.

6. Limitations

   Please provide a separate Limitations section, seems not clear.
   Authors must clearly state any limitations they see in relation to their data. Limitations may be presented in bullet point format.

**Authors’ responses:**

Dear Reviewer, thank you for your comments. We provided a separate limitation section based on your feedback in page 21, line 424.

Thank you.
