# Supplementary material for: Hematological and immunological profiles of podoconiosis patients in West Gojjam Zone, Ethiopia: A comparative cross-sectional study
Source: PLoS Negl Trop Dis. 2024 Dec 17;18(12):e0012732. doi: 10.1371/journal.pntd.0012732 (PMC11687886; doi:10.1371/journal.pntd.0012732)
Supplement: Attachment — Submitted filename: REVIEWERS COMMENTS PLOS 15-10-2024.docx [file pntd.0012732.s003.docx]

**REVIEWERS COMMENTS**

1. Abstract

Please Link your findings / results of stool samples mentioned in the abstract to the discussion section.

2. Methods: What is the clinical relevancy of ABO & Rh in podoconiosis, Malaria? Link this to discussion section.
3. Check gridline 171 typing errors (Double bracket)
4. Grid line 190 why is it in blue color
5. Grid lines 164, which blood smear type did you stain with Giemsa, and how many smears did you make because I see you also used wrights stain in another section of cell counting?
6. In discussion chapter, please relate your findings on blood grouping, stool analysis and malaria as well in relation to anemia

Thank you.
