# Supplementary material for: Hematological and immunological profiles of podoconiosis patients in West Gojjam Zone, Ethiopia: A comparative cross-sectional study
Source: PLoS Negl Trop Dis. 2024 Dec 17;18(12):e0012732. doi: 10.1371/journal.pntd.0012732 (PMC11687886; doi:10.1371/journal.pntd.0012732)
Supplement: Attachment — Submitted filename: Response to Reviewers_final.docx [file pntd.0012732.s004.docx]

**Responses to Reviewers**

**Manuscript Number: PNTD-D-24-00819)**

**Hematological and Immunological Profiles of Podoconiosis Patients in West Gojjam**

**Zone, Ethiopia: A comparative cross-sectional study**

We thank all the reviewers for their comments on this manuscript, and hope that the amendments made below do justice to the comments and improve the manuscript.

Reviewer #1: comments from the first review are addressed

Reviewer #2: (No Response)

We would like to express our gratitude to the reviewers for dedicating time to reading our manuscript and providing valuable constructive feedback that greatly contributed to the improvement of the manuscript during the revision process.

Reviewer #3: The paper has been clarified in general. However, there is still missing some relevant information.

The authors have decided not to include details about parasitological examinations because they want to present them in a separate publication. However, results seem to be incomplete or ambiguous in this case.

We appreciate the reviewer for highlighting this issue. We conducted a parasitological examination to serve as a proxy for common infectious diseases within the study population, as these could potentially affect our main outcomes (hematological and immunological parameters). We included the parasite data in our multivariate model to adjust the outcome variables, and we have noted this in the revised manuscript (page 14, lines 246-247). We do believe, though, that further discussion about the parasitological analysis in the current manuscript is beyond the scope of this study, and this variable is listed as demographic information see Table 1)

Regarding leg-washing practices, the authors state that " our data showed that podoconiosis patients wash their legs more frequently than controls. This increased frequency is likely due to the need to alleviate lymphedema-related discomfort. Frequent washing helps reduce pain and is also practical for patients who are less involved in physically demanding activities, such as farming, due to their condition."

We appreciate the reviewer's comments and acknowledge that the frequency of leg-washing practices may be influenced by the presence of lymphedema discomfort and/or serve as proxy indicators for socioeconomic status. Therefore, we have adjusted our multivariate model to account for these variables in the outcome (see the footnotes in the tables).

This apparently is a surmise or hypothesis. Authors should consider whether religion might play a role. Muslim population practice ablutions but it doesn't imply that it is a thorough wash that may remove direct contact of soil with the skin.

Thank you for picking this up. When we designed the study, we thought that religion might influence washing practices among the study population. Therefore, we included questions about religious status in our data collection questionnaire. However, in the study area where podoconiosis is prevalent, all participants, both cases and controls, were Christians. As a result, we were unable to further analyze the data (see Table 1).

Reviewer #1: most of the comments from the first review are addressed
Please again correct the misinformation which states Podoconiosis is 'classified as a Neglected Tropical 64 Disease (NTD) by the World Health Organization in 2011' ....

We thank the reviewer for this comment and apologize for this oversight. We have now modified the sentence to reflect the revised manuscript.

**REVIEWERS COMMENTS**

1. Abstract

Please Link your findings / results of stool samples mentioned in the abstract to the discussion section.

We appreciate the reviewer for highlighting this issue. We conducted a parasitological examination to serve as a proxy for common infectious diseases within the study population, as these could potentially affect our main outcomes (hematological and immunological parameters). We included the parasite data in our multivariate model to adjust the outcome variables, and we have noted this in the revised manuscript (page 14, lines 246-247). We do believe, though, that further discussion about the parasitological analysis in the current manuscript is beyond the scope of this study, and this variable is listed as demographic information (see Table 1).

2. Methods: What is the clinical relevancy of ABO & Rh in podoconiosis, Malaria? Link this to discussion section.

We thank the reviewer for their comments. We tried to address your concern regarding the clinical relevance of inclusion of ABO &Rh determination and malaria screening in our study.

**ABO blood group**

A previous study examined the possible link between ABO blood groups and cytokine levels in COVID-19 disease outcomes. This study found that higher cytokine levels in individuals with blood group O are associated with a better outcome compared to those with blood groups A, B, or AB in COVID-19 patients[1]. Therefore, we have included ABO measurements to adjust our outcome variable. We add a few sentences in the discussion section (page 20, ## Line 384-388) to reflect these comments, which reads.

*“A recent study found a link between the ABO blood group and cytokine levels in COVID-19 patient outcomes. The researchers reported that individuals with blood group O had higher cytokine levels, which were associated with better outcomes compared to those with blood groups A, B, or AB [**1**]. We also included the ABO blood group in our model and adjusted our main outcome variables accordingly.”*

**Malaria**

We also included malaria infection status in our study to account for its potential confounding effects on red cell and platelet parameters, as malaria is known to reduce red cell counts [2] and platelet counts [3]. However, none of the study participants tested positive, which prevented us from conducting further analysis. We noted this in the result section of the manuscript (Page 20 line 245-246),

3. Check gridline 171 typing errors (Double bracket)

Thanks now corrected

4. Grid line 190 why is it in blue color

Thanks now corrected

5. Grid lines 164, which blood smear type did you stain with Giemsa, and how many smears did you make because I see you also used wrights stain in another section of cell counting?

Thank you for asking for clarification on our procedures. For malaria parasite detection and identification, we stained both thick and thin blood smears with Giemsa stain, following the national guideline. We prepared 120 slides, each containing both a thick and thin smear for each participant. Additionally, we prepared 120 Wright-stained thin smears for differential white blood cell counts and morphological assessments, as Giemsa stain is not optimal for detailed blood cell morphology. We indicated this in the method section of the manuscript on page 12, lines 210-212.

In discussion chapter, please relate your findings on blood grouping, stool analysis, and malaria as well in relation to anemia

Please refer to response 2, where we address these comments.

References:

1.	Tamayo-Velasco Á, Peñarrubia Ponce MJ, Álvarez FJ, Gonzalo-Benito H, de la Fuente I, Pérez-González S, Rico L, Jiménez García MT, Sánchez Rodríguez A, Hijas Villaizan M *et al*: **Can the Cytokine Profile According to ABO Blood Groups Be Related to Worse Outcome in COVID-19 Patients? Yes, They Can**. *Front Immunol* 2021, **12**:726283.

2.	Diallo S, Roberts SA, Gies S, Rouamba T, Swinkels DW, Geurts-Moespot AJ, Ouedraogo S, Ouedraogo GA, Tinto H, Brabin BJ: **Malaria early in the first pregnancy: Potential impact of iron status**. *Clin Nutr* 2020, **39**(1):204-214.

3.	Burhan H, Hasan AS, Mansur-Ul-Haque S, Zaidi G, Shaikh T, Zia A: **Association between blood group and susceptibility to malaria and its effects on platelets, TLC, and Hb**. *J Infect Dev Ctries* 2016, **10**(10):1124-1128.
