# Supplementary material for: Hematological and immunological profiles of podoconiosis patients in West Gojjam Zone, Ethiopia: A comparative cross-sectional study
Source: PLoS Negl Trop Dis. 2024 Dec 17;18(12):e0012732. doi: 10.1371/journal.pntd.0012732 (PMC11687886; doi:10.1371/journal.pntd.0012732)
Supplement: Attachment — Submitted filename: REVIEWERS COMMENTS PLOS 20-11-2024.docx [file pntd.0012732.s005.docx]

**20-11-2024**

**REVIEWERS COMMENTS**

1. This is to acknowledge that the comments earlier raised were addressed. Please go on to help the research on the next step.

Thank you.
